# One-on-one comparison between qCSI and NEWS scores for mortality risk assessment in patients with COVID-19

**DOI:** 10.1080/07853890.2022.2042590

**Published:** 2022-02-23

**Authors:** Francisco Martín-Rodríguez, Ancor Sanz-García, Guillermo J. Ortega, Juan F. Delgado-Benito, Eduardo García Villena, Cristina Mazas Pérez-Oleaga, Raúl López-Izquierdo, Miguel A. Castro Villamor

**Affiliations:** a Unidad Móvil de Emergencias Valladolid I, Gerencia de Emergencias Sanitarias, Gerencia Regional de Salud de Castilla y León (SACYL), Valladolid, Spain; b Centro de Simulación Clínica Avanzada, Facultad de Medicina, Universidad de Valladolid, Valladolid, Spain; c Data Analysis Unit, Instituto de Investigación Sanitaria del Hospital de la Princesa (IIS-IP), Madrid, Spain; d Consejo Nacional de Investigaciones Científicas y Técnicas, CONICET, Buenos Aires, Argentina; e Unidad Móvil de Emergencias de Salamanca, Gerencia de Emergencias Sanitarias, Gerencia Regional de Salud de Castilla y León (SACYL), Valladolid, Spain; f Escuela Politécnica Superior, Universidad Europea del Atlántico, Santander, Spain; g Departamento de Medio Ambiente y Sostenibilidad, Universidad Internacional Iberoamericana, Arecibo, Puerto Rico (EE.UU); h Servicio de Urgencias, Hospital Universitario Rio Hortega de Valladolid, Gerencia Regional de Salud de Castilla y León (SACYL), Valladolid, Spain

**Keywords:** Clinical decision rules COVID-19 Risk Scores NEWS, qCSI

## Abstract

**Objective:**

To compare the predictive value of the quick COVID-19 Severity Index (qCSI) and the National Early Warning Score (NEWS) for 90-day mortality amongst COVID-19 patients.

**Methods:**

Multicenter retrospective cohort study conducted in adult patients transferred by ambulance to an emergency department (ED) with suspected COVID-19 infection subsequently confirmed by a SARS-CoV-2 test (polymerase chain reaction). We collected epidemiological data, clinical covariates (respiratory rate, oxygen saturation, systolic blood pressure, heart rate, temperature, level of consciousness and use of supplemental oxygen) and hospital variables. The primary outcome was cumulative all-cause mortality during a 90-day follow-up, with mortality assessment monitoring time points at 1, 2, 7, 14, 30 and 90 days from ED attendance. Comparison of performances for 90-day mortality between both scores was carried out by univariate analysis.

**Results:**

From March to November 2020, we included 2,961 SARS-CoV-2 positive patients (median age 79 years, IQR 66–88), with 49.2% females. The qCSI score provided an AUC ranging from 0.769 (1-day mortality) to 0.749 (90-day mortality), whereas AUCs for NEWS ranging from 0.825 for 1-day mortality to 0.777 for 90-day mortality. At all-time points studied, differences between both scores were statistically significant (*p* < .001).

**Conclusion:**

Patients with SARS-CoV-2 can rapidly develop bilateral pneumonias with multiorgan disease; in these cases, in which an evacuation by the EMS is required, reliable scores for an early identification of patients with risk of clinical deterioration are critical. The NEWS score provides not only better prognostic results than those offered by qCSI at all the analyzed time points, but it is also better suited for COVID-19 patients.

**KEY MESSAGES**
- This work aims to determine whether NEWS is the best score for mortality risk assessment in patients with COVID-19.
- AUCs for NEWS ranged from 0.825 for 1-day mortality to 0.777 for 90-day mortality and were significantly higher than those for qCSI in these same outcomes.
- NEWS provides a better prognostic capacity than the qCSI score and allows for long-term (90 days) mortality risk assessment of COVID-19 patients.

## Introduction

### Background

In its initial stages back in February-March 2020, the current coronavirus disease 2019 (COVID-19) pandemic was a global shock to health systems all around the world [1]. In the course of time, the scientific community has developed procedures to adequately screen and manage the huge number of patients generated by the *severe acute respiratory syndrome coronavirus 2* (SARS-CoV-2) [2,3].

Over the course of the pandemic, several challenges have been overcome in record time, remarkably, early virus detection techniques (antibody and antigen testing) and mass vaccination [4,5]. Nevertheless, the early identification of patients at high-risk of clinical deterioration is still being refined. Initially, patient categorization was complex and early warning scores already implemented in health systems were used, even if they were not specifically designed for COVID-19, as was the case for instance of the National Early Warning Score (NEWS) [6,7].

### Importance

Throughout the pandemic, specific scores have been developed to assess disease severity in COVID-19 patients [8–10]. The best performing scores have in common the use of laboratory or imaging variables (C-reactive protein, urea, leukocytes, chest computed tomography) not always available in certain healthcare environments [11,12]. Therefore, new simple and easily applicable scores capable of providing relevant risk discrimination capacity have been developed, such as the quick COVID-19 Severity Index (qCSI) [13].

COVID-19 causes an increase in primary care centre consultations, ambulance transfers, emergency department visits, hospital and intensive care unit admissions, and finally, an increase in unexpected mortality. Severe cases present with bilateral pneumonias accompanied by multiorgan disease in which the ventilatory function is especially compromised [14]. Under such circumstances, the early identification of cases at risk of deterioration in the short-term is critical for the strategic management of the pandemic. It is necessary to objectively prioritize those patients with higher probability of survival amongst those at higher risk; at this crossroads, early warning scores can help in the decision-making process [15,16]. Both the qCSI and the NEWS scores include, with different weights, parameters related to the ventilatory function (respiratory rate, oxygen saturation and supplemental oxygen administration), which is critical in the early detection of the risk of deterioration in patients with COVID-19 [7,17,18]. Until now, however, no direct comparison has been done between both scores. This comparison seems mandatory since NEWS is considered the gold standard of early warning scores, and such comparison could determine the added value of the new score.

### Goals of this investigation

The present study aims to compare the predictive values of the qCSI and the NEWS scores for 90-day mortality amongst patients with COVID-19 transferred to the emergency department by emergency medical services

## Methods

### Study population

The inclusion criteria were as follows: Adult patients (>18 years) with COVID-19 infection confirmed by SARS-CoV-2 test (polymerase chain reaction) and transferred by ambulance to EDs. The exclusion criteria included: patients under 18 years of age, without analytical confirmation of infection, or cases in which the lack of any variable impeded the estimation of the scores analyzed.

### Study design

This was a multicenter retrospective cohort study analyzing health data from March to November 2020, from an overall reference population of 1,166,408 inhabitants. The study was carried out in the provinces of Palencia, Salamanca, Segovia and Valladolid (Spain) with the participation of 61 ambulance services and EDs from 8 hospitals (three tertiary university hospitals and five general district hospitals). Both the Emergency Medical Services (EMS) and the hospitals are managed by the Public Health System of Castilla-León (SACYL), the principal health operator.

The study was approved by the local institutional research review board of Rio Hortega Hospital (PI 138/20) and conformed to the Declaration of Helsinki principles. The institutional review board granted a waiver of the obligation to collect consent from study participants due to the use of unidentified subjects.

### Outcome

The outcome was cumulative all-cause mortality during a 90-day follow-up, with mortality assessment at the following monitoring time points: 1, 2, 7, 14, 30 and 90 days from ED attendance. All monitoring time points were also cumulative, i.e. deaths registered at a particular point also include those registered at the preceding ones, e.g. 14-day mortality includes also patients of the preceding time points 1, 2, 3 and 7 days.

### Predictors and data abstraction

Epidemiological data (sex, age, rural or urban area and nursing home origin) and clinical covariates (respiratory rate, oxygen saturation, systolic blood pressure, heart rate, temperature, level of consciousness and use of supplemental oxygen) were collected by an emergency registered nurse at the triage emergency room, so these variables were blinded to the clinical investigators.

The Connex^®^ Vital Signs Monitor (Welch Allyn, Inc., Skaneateles Falls, NY) was used to measure blood pressure, heart rate, temperature and oxygen saturation. The respiratory frequency was measured by direct observation of the respiratory cycles for 30 s; in case of irregular breathing or extreme ranges, it was measured by direct auscultation for 1 min. The level of consciousness was assessed by means of the Glasgow Coma Scale; a score of less than 15 was considered alteration of the level of consciousness. Finally, the percentage of oxygen was evaluated by means of the fraction of inspired oxygen supplied, from which the litres per minute of oxygen administered were calculated.

Ninety days from the first care, an independent clinical investigator from each hospital reviewed the patient's electronic medical record and collected mortality data, destination (discharge on site, admission for hospitalization or intensive care unit) and 17 categories of comorbidities necessary to calculate the Charlson comorbidity index.

The NEWS and qCSI scores were calculated using vital sign data and clinical observations in accordance with the Royal College of London standards for NEWS (note that NEWS or NEWS2 were used indistinctly, and both refer to NEWS2) [19,20] and Haimovich et al. [13] for qCSI (see Supplementary Data S1 and S2). In particular, values for both scores were calculated with physiological measurements obtained from the electronic medical record (collected on the patient's arrival at the ED). Mortality data at 1, 2, 3, 7, 14, 30 and 90 days were also obtained from the electronic health records. The score values were subsequently used to analyze the predictive capacity of both scores for mortality at the different time points.

### Primary data analysis

Categorical variables were represented by absolute values and percentages. Continuous variables were represented by median and interquartile range (IQR) since they did not follow a normal distribution. For the characterization of the total sample and to analyze the association between each independent variable and the primary outcome (90-day mortality), the Mann-Whitney *U* test or chi-squared test was performed as appropriate.

The discriminatory validity of the scores was assessed by the area under the receiver operating characteristic (ROC) curve (AUC), calculating in each case the *p* value of the hypothesis contrast. The graphs of the ROC curves show the confidence interval (95% CI) obtained by resampling (or bootstrapping) 2000 realizations. Finally, the specificity, sensitivity, positive predictive value, negative predictive value, positive likelihood ratio and negative likelihood ratio of the score obtained were calculated. With the objective of comparing ROCs, a Delong’s test and a decision curve analysis were used. The effect of confounding factors (i.e. age, sex, and comorbidities, evaluated by the Charlson Age Comorbidity Index [CACI]) on the predictive value of both scores was assessed by a multivariate analysis. Data were analyzed using our own codes and base functions in R, version 4.0.3 (http://www.R-project.org; the R Foundation for Statistical Computing, Vienna, Austria).

## Results

### Baseline characteristics

A total of 3183 patients met the inclusion criteria. After applying the exclusion criteria, the final sample for analysis consisted of 2961 patients with confirmed SARS-CoV-2 infection (see Supplementary Figure S1).

Patients were mostly older adults (median age 79 years, IQR 66-88, range 18–104), with an almost uniform gender balance (49.2% females). The hospitalization rate was 78.6%, with 5.5% requiring intensive care unit (ICU) admission. The cumulative all-cause mortality at the monitoring time points 1, 2, 7, 14, 30 and 90 days was 5.7% (169 cases), 8.2% (243 cases), 18.1% (537 cases), 24.2% (718 cases), 27.9% (827 cases) and 32% (948 cases), respectively (Table 1).

**Table 1.** Demographic and clinical characteristics for 90-day mortality.

		90-day mortality		
Characteristics^a^	Total No.	Non-survivors	Survivors	*p* value
No. with data	2961	948 (32)	2013 (68)	
Age (years)	79 (66–88)	86 (79–90)	74 (60–85)	<.001
<50	260 (8.8)	10 (1.1)	250 (12.4)	
50–65	460 (15.5)	36 (3.8)	424 (21.1)	<.001
66–79	774 (26.1)	194 (20.5)	580 (28.8)	<.001
>80	1467 (49.5)	708 (74.7)	759 (37.7)	<.001
Sex, female	1457 (49.2)	439 (46.3)	1018 (50.6)	.030
Urban area	1551 (52.4)	473 (49.9)	1078 (53.6)	.063
Nursing homes	1080 (36.5)	526 (55.5)	554 (27.5)	<.001
Basal evaluation				
RR (breaths/min)	16 (13–25)	24 (14–26)	14 (12–22)	<.001
SpO_2_ (%)	94 (90–96)	90 (84–95)	95 (92–97)	<.001
FiO_2_ (%)	0.21 (0.21–0.21)	0.21 (0.21–0.28)	0.21 (0.21–0.21)	<.001
SBP (mmHg)	126 (111–144)	123 (106–144)	127 (114–144)	<.001
Heart rate (beats/min)	87 (76–100)	89 (75–104)	86 (76–98)	.001
Temperature (°C)	36.6 (36.2–37.3)	36.7 (36.1–37.4)	36.6 (36.2–37.2)	.207
GCS (points)	15 (15-15)	15 (13–15)	15 (15-15)	<.001
qCSI-19 SI (points)	1 (1–5)	5 (1–6)	0 (0–2)	<.001
NEWS (points)	4 (2–8)	8 (5–10)	3 (2–6)	<.001
CCI (points)	1 (1–3)	2 (1-4)	1 (1–3)	<.001
AIDS	5 (0.2)	2 (0.2)	3 (0.1)	.702
Metastatic disease	42 (1.4)	22 (2.3)	20 (1)	.004
Liver disease severe	75 (2.5)	19 (2)	56 (2.8)	.209
Lymphoma	17 (0.6)	6 (0.6)	11 (0.5)	.772
Leukemia	38 (1.3)	12 (1.3)	26 (1.3)	.954
Solid tumour localized	383 (12.9)	130 (13.7)	253 (12.6)	0.387
DM end organ damage	147 (5)	67 (7.1)	80 (4)	<.001
Severe CKD	454 (15.3)	203 (21.4)	251 (12.5)	<.001
Hemiplegia	87 (2.9)	48 (5.1)	39 (1.9)	<.001
DM uncomplicated	566 (19.1)	197 (20.8)	369 (18.3)	.114
Liver disease mild	89 (3)	28 (3)	61 (3)	.909
Peptic ulcer	84 (2.8)	34 (3.6)	50 (2.5)	.092
Connective	92 (3.1)	29 (3.1)	63 (3.1)	.918
COPD	310 (10.5)	117 (12.3)	193 (9.6)	.022
Dementia	719 (24.3)	385 (40.6)	334 (16.6)	<.001
Cerebrovascular disease	322 (10.9)	156 (16.5)	166 (8.2)	<.001
Peripheral vascular disease	255 (8.6)	100 (10.5)	155 (7.7)	.010
Congestive heart failure	437 (14.8)	190 (20)	247 (12.3)	<.001
Myocardial infarction	280 (9.5)	119 (12.6)	161 (8)	<.001
Outcomes				
Hospitalization	2328 (78.6)	901 (95)	1427 (70.9)	<.001
ICU	162 (5.5)	73 (7.7)	89 (4.4)	<.001

Figures represent the descriptive statistics and *p* value of the comparison between non-survivors and survivors.

RR: Respiratory rate; SpO_2_: pulse oximetry saturation; FiO_2_: fraction of inspired oxygen; GCS: Glasgow coma scale; qCSI-19 SI: quick COVID-19 Severity Index; NEWS: National Early Warning Score; CCI: Charlson comorbidity index; AIDS: acquired immunodeficiency syndrome; DM: Diabetes mellitus; CKD: chronic kidney disease; COPD: chronic obstructive pulmonary disease; ICU: intensive care unit.

^a^Values expressed as total number (fraction) and medians [25–75‰], as appropriate.

### Scores’ discrimination

**Figure 1.**
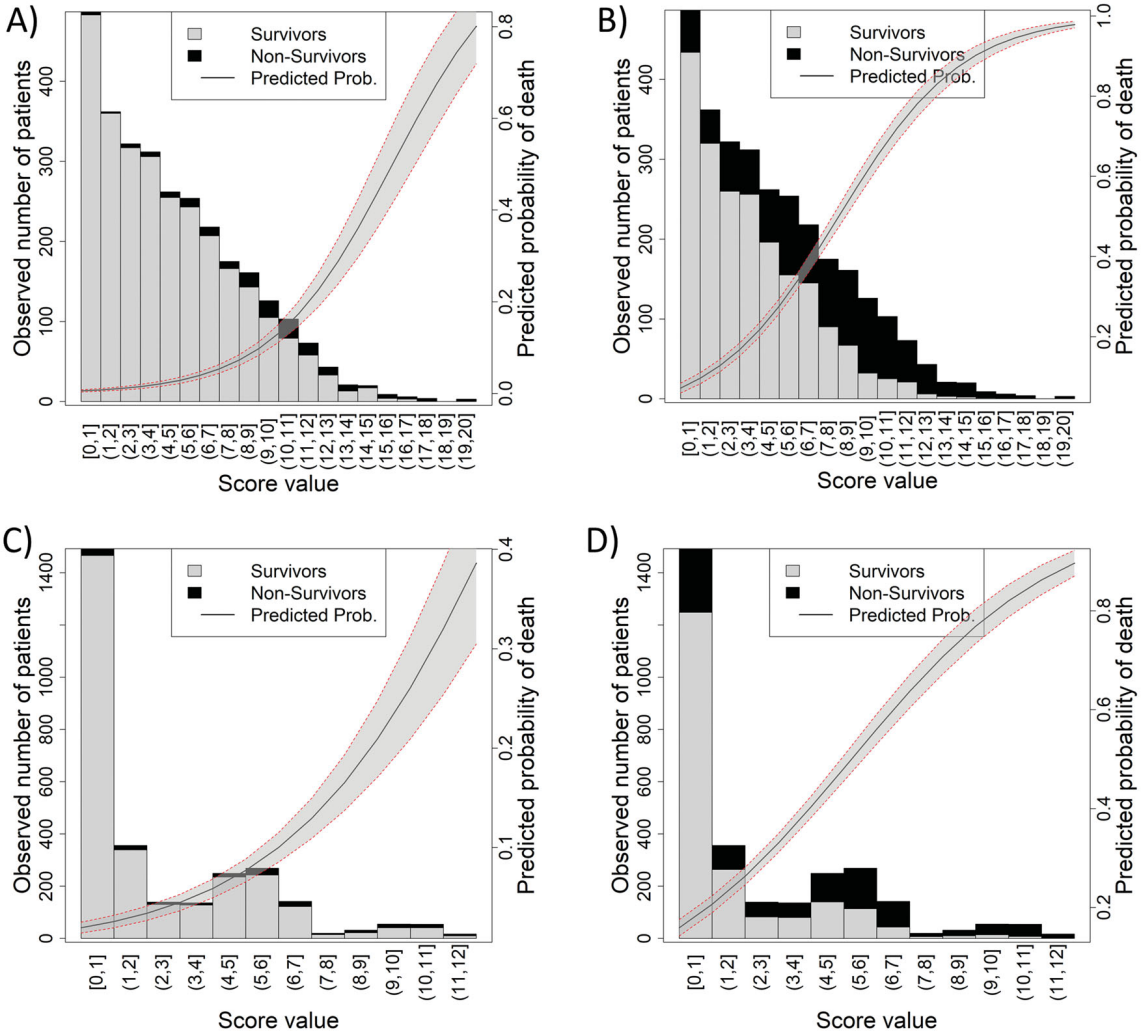
Predicted probability of death and observed distribution of patients across score value of NEWS for 1-day (A) and 90-day mortality (B) and qCSI for 1-day (C) and 90-day mortality (D). The grey area of the trend line corresponds to 95% confidence interval of the predicted probability of death (trend line). The bars correspond to the number of patients alive (grey) or dead (black) in the training cohort. The values within parenthesis refer to the range of score values included in each bar.

The discrimination capacity of each score for 1-day and 90-day mortality was assessed analyzing the distribution of survivors and non-survivors and the predicted probability of mortality according to different values of the score (Figure 1). For NEWS, higher score values included a higher proportion of non-survivors and predicted a higher probability of mortality, both for 1-day (Figure 1(A)) and 90-day (Figure 1(B)) mortality. Moreover, lower score values predicted higher probability of death at 90 days than at 1 day. qCSI showed a similar distribution for 1-day (Figure 1(C)) and 90-day (Figure 1(D)) mortality, although it provided lower probabilities of death than NEWS throughout the whole range of score values. Similar discrimination results at the other time points (2, 7, 14 and 30 days) can be found in the Supplementary Data S3.

### Scores comparison

**Figure 2.**
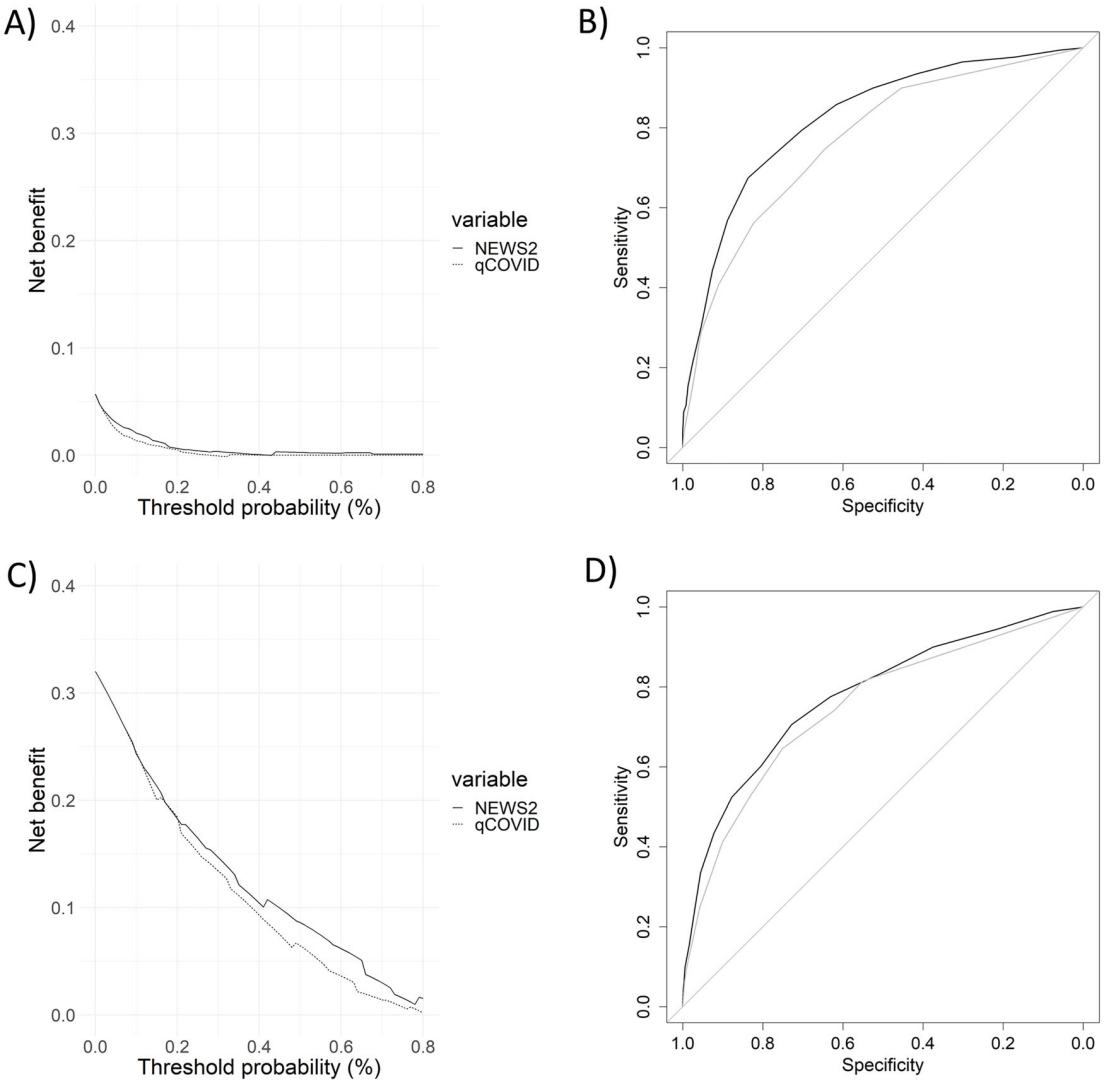
Predictive validity results of each model. Decision curve analysis for 1-day (A) and 90-day mortality (C) and the area under the receiver operating characteristic (ROC) curves for 1-day (B) and 90-day mortality (D) of qCSI and NEWS. Grey line corresponds to qCSI results and the black line to NEWS results.

The predictive capacity of each score for mortality was assessed by ROC curves analysis and by decision curves. The comparison of results for both scores for mortality at 1 day and 90 days is shown in Figure 2. Similar comparisons were carried out for the intermediate time points (Supplementary Data S4). Although AUCs decreased with increasing times for both scores, AUC values were higher for the NEWS score than for qCSI throughout the whole range of time points (Table 2), indicating that NEWS has a better performance for mortality prediction. Finally, regarding statistical parameters related to AUCs, NEWS showed better sensitivity, positive predictive value, negative predictive value and positive likelihood ratio than qCSI, whereas its specificity and negative likelihood ratio were worse (Table 3).

**Table 2.** Predictive validity of qCSI and NEWS for different mortality time points.

	1-day	2-day	7-day	14-day	30-day	90-day
NEWS	0.825	0.823	0.792	0.780	0.779	0.777
qCSI	0.769	0.782	0.760	0.745	0.750	0.749
*p* Value	<.001	<.001	<.001	<.001	<.001	<.001

Figures represent the area under the receiver operating characteristic (ROC) curve (AUC) for each score, and the p value (Delong’s test) of the AUCs comparison at each time point.

NEWS: National Early Warning Score; qCOVID: quick COVID-19 Severity Index.

**Table 3.** Statistical details of the qCSI and NEWS for different point times analyzed.

Mortality	Specificity	Sensitivity	PPV	NPV	LR +	LR −
1-day						
NEWS	72.1 (56.5–87.5)	51.5 (33.7–69.2)	32.4 (18.2–46.7)	96.7 (95.8–97.6)	8.94 (1.95–15.9)	0.55 (0.39–0.71)
qCSI	76.1 (59.6–92.4)	47.9 (28.6–67.2)	19.2 (12.9–25.5)	96.3 (95.4–97.2)	4.29 (2.48–6.09)	0.63 (0.47–0.78)
2-day						
NEWS	72.5 (57.1–88)	50.4 (32.5–68.2)	39.2 (24.8–53.7)	95.2 (93.9–96.5)	10.5 (2.04–19.1)	0.56 (0.4–0.72)
qCSI	76.7 (60.3–93.1)	48.4 (29–67.8)	27.7 (19–36.5)	94.8 (93.5–96.1)	4.96 (2.78–7.13)	0.61 (0.45–0.77)
7-day						
NEWS	74.1 (58.8–89.5)	45.1 (27.4–62.9)	53.1 (40.6–65.7)	88.1 (85.1–90.7)	6.76 (3.29–10.2)	0.63 (0.47–0.78)
qCSI	78.5 (62.4–94.9)	43.4 (24.1 (62.8)	48 (36.2–59.8)	87.1 (84.6–89.7)	5.93 (2.54–9.33)	0.67 (0.52–0.82)
14-day						
NEWS	75.1 (59.8–90.2)	43.1 (25.4–60.6)	60.3(48.5–72.1)	83.2 (79.8–86.5)	7.5 (2.97–12.03)	0.65 (0.5–0.8)
qCSI	79.6 (63.5–95.7)	40.7 (21.5–59.8)	56.1 (44.4–67.7)	81.8 (78.8–84.9)	6.16 (2.42–9.91)	0.7 (0.56–0.84)
30-day						
NEWS	75.6 (60.5–90.8)	42.2 (24.7–59.7)	65.3 (53.5–77.1)	80.2 (76.4–83.9)	6.96 (3.22–10.6)	0.66 (0.52–0.81)
qCSI	80.3 (64.3–96.3)	39.9 (20.6–59.1)	60.4 (49.6–71.2)	78.8 (75.3–82.3)	5.8 (2.71–8.89)	0.7 (0.56–0.85)
90-day						
NEWS	76.3 (61.2–91.4)	41.3 (23.9–58.7)	69.7 (58.4–81.1)	76.7 (72.6–80.7)	9.3 (3.19–15.4)	0.67 (0.53–0.81)
qCSI	81.1 (65.1–97)	38.8 (19.7–57.9)	65.2 (54.7–75.6)	75.1 (71.3–79)	5.85 (3.08–8.62)	0.71 (0.57–0.85)

Figures represent different metrics derived from the area under the receiver operating characteristic (ROC) curve (AUC) for each score at each time point.

NEWS: National Early Warning Score; qCSI: quick COVID-19 Severity Index; PPV: positive predictive value; NPV: negative predictive value; LR: likelihood ratio.

Bracketed number indicate 95% confidence interval.

Finally, we carried out a multivariate analysis to assess the effect of putative confounding variables such as age, sex and comorbidities (CACI) on the predictive ability of both scores for mortality (Supplementary Data 5). For short mortality times (1 and 2 days) no effect was found. For 7-day mortality, being >89 years was a risk factor for both NEWS and qCSI and being male for NEWS. For longer times all confounding variables were found as risk factors for mortality.

## Discussion

In the present study, the NEWS score showed a better ability to predict mortality in patients with COVID-19 than the qCSI score at all-time points analyzed during a 90-day follow-up period.

In an emergency situation, such as the current COVID-19 pandemic, it is critical to accurately predict the risk of clinical deterioration in order to balance provisions of life-saving care and to steward precious resources [14,21]. In this sense, early warning scores are reliable tools in such critical decisions [22]. For instance, NEWS is commonly used in several clinical situations [23–26] and has been validated for COVID-19 [7] . The qCSI, instead, was only internally validated [13], and there is limited experience in its implementation [27].

In the cohort of the present study, NEWS had an AUC of 0.825 for 1-day mortality, in line with recent studies [16,17,28,29], performing better than qCSI, which showed an AUC of 0.769. In the study by Haimovich et al. [13], where qCSI was established and validated, AUC of this score was 0.82, though the cohort used was smaller than ours. Importantly, in our study, the performance of both scores was not affected by age, sex or comorbidities up to 2 days, revealing their short-term potential. However, at longer mortality times, from 7 to 90 days, all the confounding factors were significantly related to mortality. This result was somehow expected, since at shorter times, initial deterioration of the patients may weigh more than intrinsic factors such as age sex and comorbidities.

Severe cases of SARS-CoV-2 are characterized by significant compromise of ventilatory function with breathlessness, desaturation and tachypnoea often requiring the administration of supplemental oxygen or invasive mechanical ventilation [30]. Both scores account (although with different weights) for ventilatory measurements, but NEWS additionally includes the cardio-vascular function (systolic blood pressure and heart rate), neurological function (Glasgow coma scale), and temperature. The addition of these parameters, a priori, makes it a more complete score than qCSI. On the other hand, unlike NEWS, qCSI focuses only on the analysis of ventilatory function, which could be seen as an advantage, especially considering that it only requires three easy-to-obtain variables.

In critical circumstances with a volume of patients exceeding the operational capabilities of health systems, an appropriate selection of those cases with the highest risk of clinical deterioration is mandatory. In this sense, qCSI is a simple score, easy to apply, but with a worse performance than NEWS. However, NEWS has been implemented in multiple health systems, tested in a wide variety of clinical contexts, and has a high prognostic performance [31–34]. On the other hand, NEWS requires collection of more variables than the qCSI, an issue that should be considered a handicap during the pandemic. In short, both scores have strengths and weaknesses. However, based on more comprehensive scientific evidence, its implementation in multiple clinical environments, its robustness from a statistical point of view, and its better prognostic performance, NEWS could be considered a better suited score than qCSI for the EDs. This statement should be considered however with the warning message that this was a retrospective study; NEWS requires additional efforts [18,29,35,36], and it was not developed exclusively for COVID-19 patients.

During the first and second waves of the ongoing COVID-19 pandemic the initial patient/resource ratio was very high. This overload revealed the necessity of appropriate triage systems to apply the limited resources to critical patients. For instance, being classified as a high-risk patient (NEWS ≥ 7) is a strong predictor of early clinical impairment. Therefore, the use of scoring systems, such as NEWS, is critical to assist in initial triage, both at the scene and in the ED, helping to manage hospital and ICU admissions in a more efficient way, and to guide decisions on transfer to the ED. Nevertheless, its usefulness goes beyond this, because at extraordinary times like the present, it will allow us to know with certainty and in a simple way the short-term vital perspective of the patient. The availability of these scores will help the health system to better organize and manage limited resources, and also will allow us to inform the relatives of seriously ill patients of the possible outcome.

This study has several limitations. First, this is a retrospective study carried out during a pandemic. Due to the extreme burden of care at certain stages, the completion of clinical histories could have been compromised with an increase in the amount of missing data. To avoid bias, a multicenter study with a significant number of patients was planned. Second, due to the multiplicity of existing scores, a partial selection was made. Of all the early warning scores, NEWS is the one supported by the most scientific evidence, the highest degree of implementation, and by applications in diverse clinical contexts and pathologies including patients with COVID-19 [7,16,17]. Third, we avoided using scores that require analytical inputs (e.g. CURB-65) or parameters that are complex to quantify during the first care. Fourth, it should be highlighted that this study was developed by considering only those patients requiring EMS, which are arguably in a poorer clinical state as compared with those non-evacuated by ambulance. Finally, qCSI grounded its prognostic capacity on early clinical worsening during the first 24 h (necessity of high-flow oxygen, invasive or non-invasive mechanical ventilation, and death), whereas in the present study 90-day mortality was taken as the primary outcome variable. To solve this, we included 1-day mortality as an outcome, because qCSI was established and validated at this time point [13].

In summary, patients with SARS-CoV-2 infection can rapidly evolve to bilateral pneumonias with multiorgan disease; in these cases, an early identification of patients at high-risk of clinical deterioration should be prioritized. The NEWS score outperformed the qCSI score in predicting mortality at all studied time points, from 1 day to 90 days. The standardized use of early warning scores in patients evacuated by the EMS can aid in the complex decision-making process, assisting healthcare workers in the initial identification of the most severe patients and supporting the best allocation of resources.

## Transparency declaration

The corresponding author on behalf of the other authors guarantee the accuracy, transparency and honesty of the data and information contained in the study, that no relevant information has been omitted and that all discrepancies between authors have been adequately resolved and described.

The following work has not been previously published and is not under consideration by any other scientific journal.

## Supplementary Material

Supplemental Material

## Data Availability

Data are available upon a reasonable request to the corresponding author in https://figshare.com/s/445db8346441bd09aba3.

## Author statement

Drs Martín-Rodríguez and López-Izquierdo had full access to all of the data in the study and take responsibility for the integrity of the data and the accuracy of the data analysis. *Study concept and design:* All authors. *Acquisition, analysis, or interpretation of data:* Delgado Benito, Mazas Pérez-Oleaga and García Villena. *Drafting of the manuscript:* Martín-Rodríguez and Sanz-García. *Final approval, critical revision of the manuscript for important intellectual content:* All authors. *Statistical analysis:* Sanz García and Ortega Rabbione. *Obtained funding:* Martín-Rodríguez. *Administrative, technical, or material support:* Mazas Pérez-Oleaga and García Villena. *Study supervision:* Castro Villamor. All authors agree to be accountable for all aspects of the work. Drs Martín-Rodríguez and López-Izquierdo are joint first authors.
