# Supplementary material for: One-on-one comparison between qCSI and NEWS scores for mortality risk assessment in patients with COVID-19
Source: Ann Med. 2022 Feb 23;54(1):646–54. doi: 10.1080/07853890.2022.2042590 (PMC8881067; doi:10.1080/07853890.2022.2042590)
Supplement: Supplemental Material [file IANN_A_2042590_SM5152.zip › suppl_data/eFigure1.docx]

**Figure 1. Study flowchart.**

Retrospective data collection

(March-November 2020)

Assessed for enrollment (n=3526)

Excluded (n=343)

- Patients < 18 years (n=37)

- Unknown identity (n=139)

- Missing data (n=167)

Fulfilled inclusion criteria (n=3183)

Excluded (n=222)

- Duplicate encounters (n=109)

- Lost to follow-up (n=113)

Patients included final cohort (n=2961)
