# Supplementary material for: One-on-one comparison between qCSI and NEWS scores for mortality risk assessment in patients with COVID-19
Source: Ann Med. 2022 Feb 23;54(1):646–54. doi: 10.1080/07853890.2022.2042590 (PMC8881067; doi:10.1080/07853890.2022.2042590)
Supplement: Supplemental Material [file IANN_A_2042590_SM5152.zip › suppl_data/supp data1.docx]

**Supplementary Table 1. National Early Warning Score 2 (NEWS2).**

| **NEWS2** | **3** | **2** | **1** | **0** | **1** | **2** | **3** |
| --- | --- | --- | --- | --- | --- | --- | --- |
| Pulse (bpm) | ≤40 |  | 41–50 | 51–90 | 91–110 | 111–130 | ≥131 |
| BR (bpm) | ≤8 |  | 9–11 | 12–20 |  | 21–24 | ≥25 |
| T (°C) | ≤35 |  | 35.1–36 | 36.1–38 | 38.1–39 | ≥39.1 |  |
| SBP (mmHg) | ≤90 | 91–100 | 101–110 | 111–219 |  |  | ≥220 |
| SpO_2_ (%) Scale 1 | ≤91 | 92–93 | 94–95 | ≥96 |  |  |  |
| SpO_2_ (%) Scale 2 ^1^ | ≤83 | 84–85 | 86–87 | 88–92  ≥93 air | 93–94 Oxygen | 95–96 Oxygen | ≥97 Oxygen |
| Air oxygen |  | Oxygen |  | Air |  |  |  |
| AVPU (scale) |  |  |  | A |  |  | V, P, U |

^1^ In patients with hypercapnic respiratory insufficiency, scale 2 should be used to weight the oxygen saturation score. SBP: Systolic blood pressure; BR: Breathing rate; SpO_2_: Oxygen saturation; AVPU: Alert, verbal, pain, unresponsive: T: Temperature. Reproduced from The Royal College of Physicians. National Early Warning Score (NEWS) 2: Standardizing the assessment of acute-illness severity in the NHS. Updated report of a working party. London: RCP, 2017.
