# Supplementary material for: One-on-one comparison between qCSI and NEWS scores for mortality risk assessment in patients with COVID-19
Source: Ann Med. 2022 Feb 23;54(1):646–54. doi: 10.1080/07853890.2022.2042590 (PMC8881067; doi:10.1080/07853890.2022.2042590)
Supplement: Supplemental Material [file IANN_A_2042590_SM5152.zip › suppl_data/supp data2.docx]

**Supplementary Table 2. Quick COVID-19 Severity Index**

| **qCSI variable** | | **points** |
| --- | --- | --- |
| BR (bpm) | |  |
|  | ≤22 | 0 |
|  | 23-28 | 1 |
|  | >28 | 2 |
| SpO_2_ (%) | |  |
|  | >92 | 0 |
|  | 89-92 | 2 |
|  | ≤88 | 5 |
| Oxygen flow rate, L/min | |  |
|  | ≤2 | 0 |
|  | 3-4 | 4 |
|  | 5-6 | 5 |

BR: Breathing rate; SpO2: Oxygen saturation
