# Supplementary material for: One-on-one comparison between qCSI and NEWS scores for mortality risk assessment in patients with COVID-19
Source: Ann Med. 2022 Feb 23;54(1):646–54. doi: 10.1080/07853890.2022.2042590 (PMC8881067; doi:10.1080/07853890.2022.2042590)

The discrimination of each score at 2-days mortality

a) NEWS


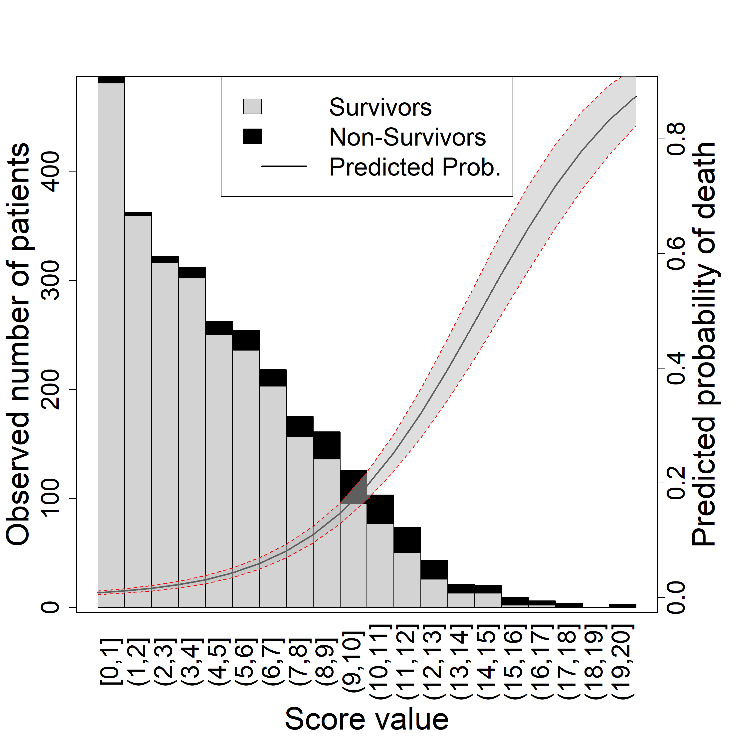


b) qCOVID


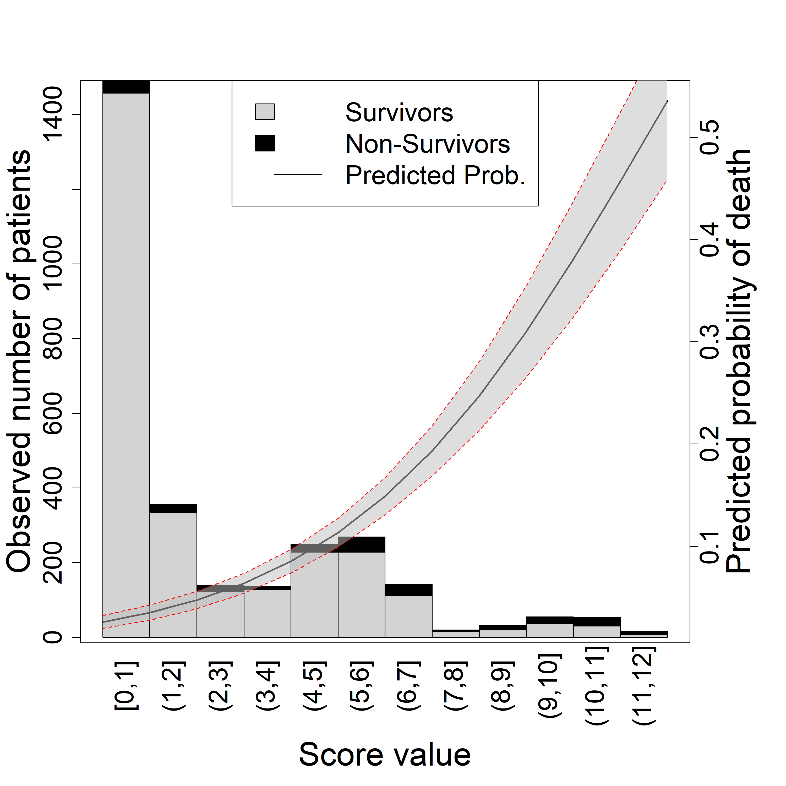


The discrimination of each score at 7-days mortality

a) NEWS


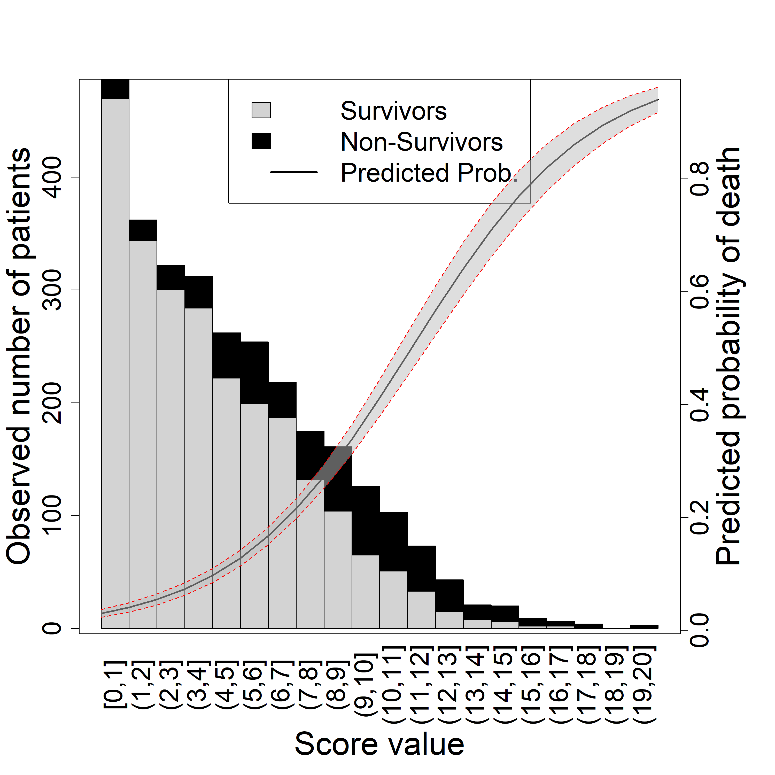


b) qCOVID


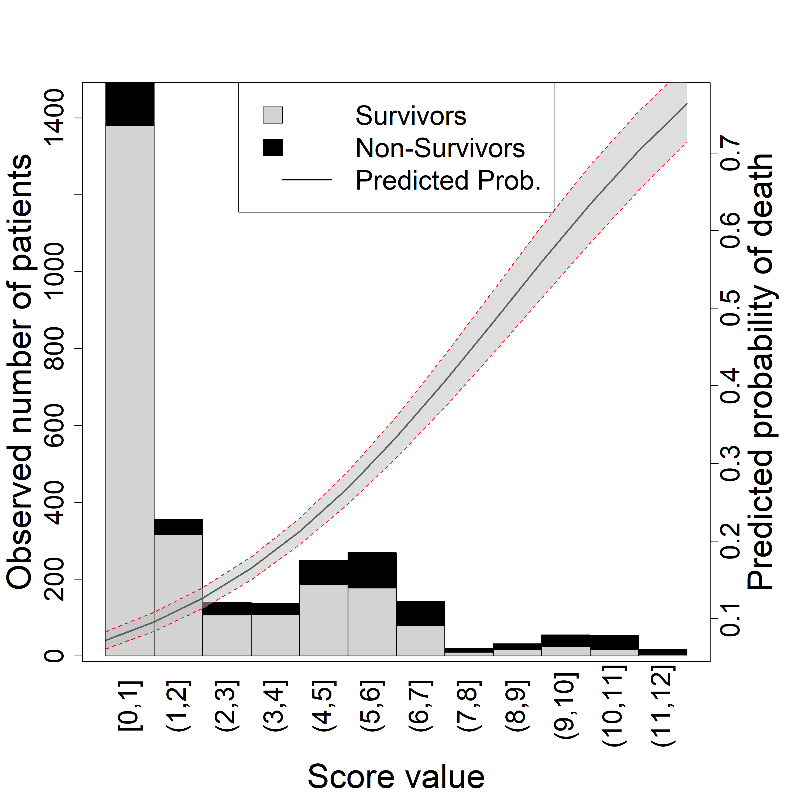


The discrimination of each score at 14-days mortality

a) NEWS


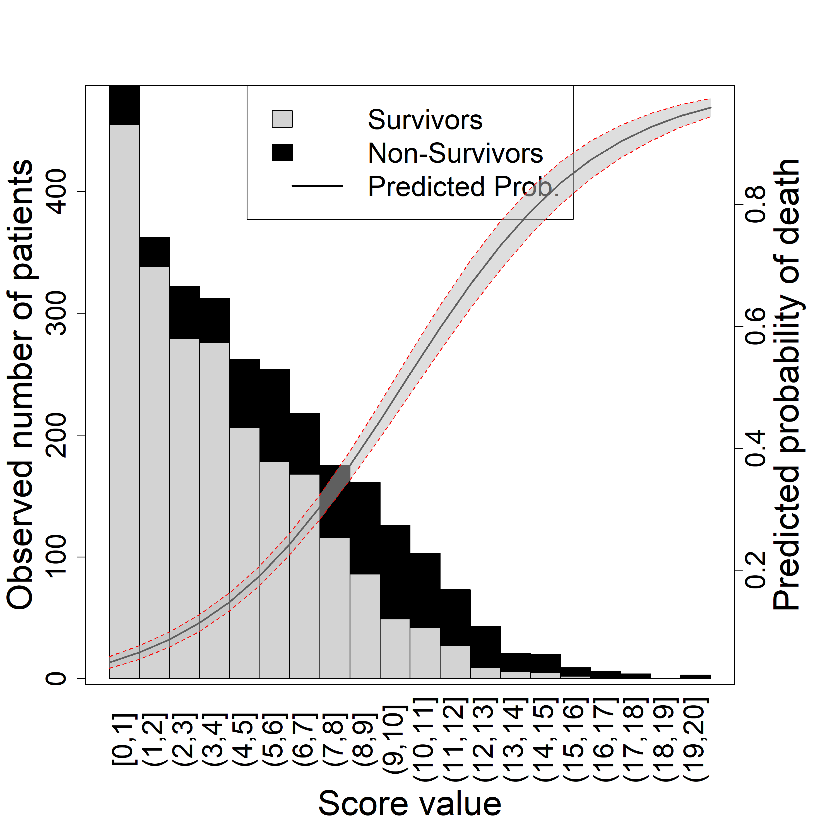


b) qCOVID


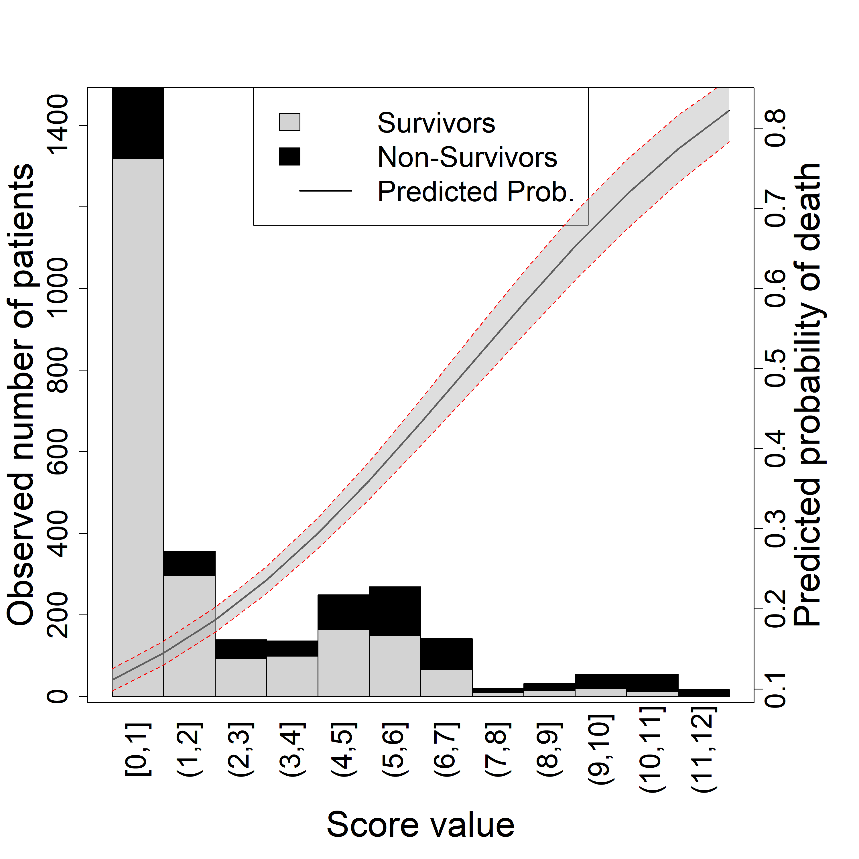


The discrimination of each score at 30-days mortality

a) NEWS


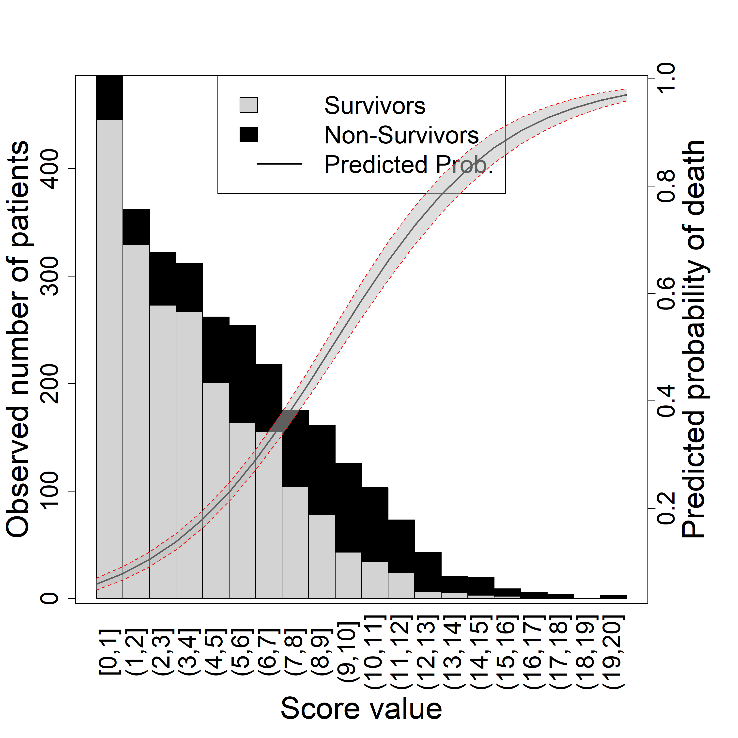


b) qCOVID


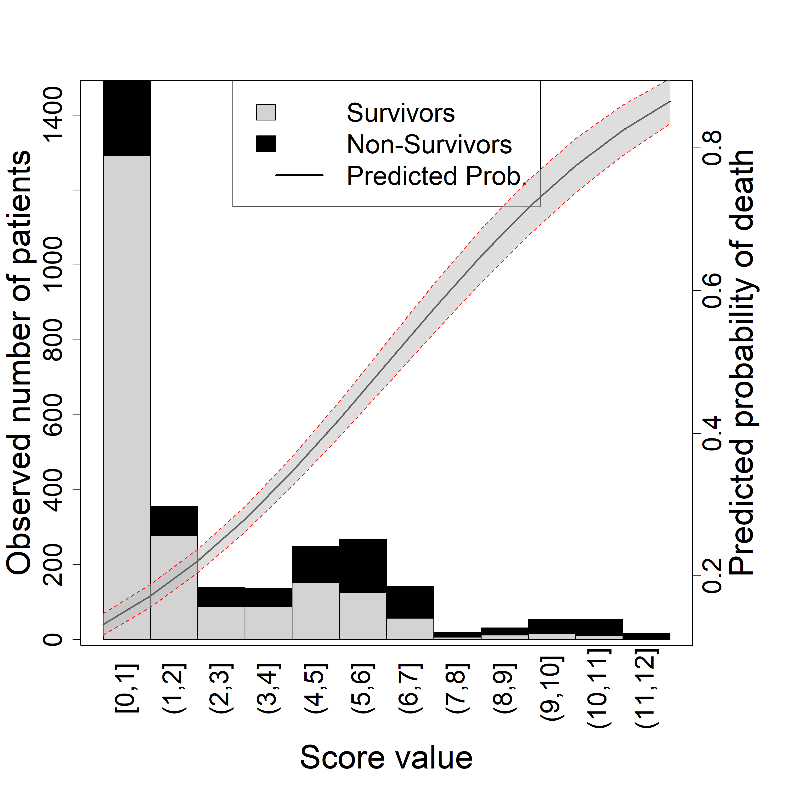

Supplement: Supplemental Material [file IANN_A_2042590_SM5152.zip › suppl_data/supp data3.docx]
