# Supplementary material for: One-on-one comparison between qCSI and NEWS scores for mortality risk assessment in patients with COVID-19
Source: Ann Med. 2022 Feb 23;54(1):646–54. doi: 10.1080/07853890.2022.2042590 (PMC8881067; doi:10.1080/07853890.2022.2042590)
Supplement: Supplemental Material [file IANN_A_2042590_SM5152.zip › suppl_data/supp data5.docx]

**Multivariate analysis for NEWS2, age, sex and Charlson Age-Comorbidity Index at 1-days mortality**

Coefficients:

Estimate Std. Error z value Pr(>|z|)

(Intercept) -6.41729 1.01648 -6.313 2.73e-10 ***

NEWS2 0.29827 0.02353 12.679 < 2e-16 ***

Sex1 0.04709 0.17786 0.265 0.791

Age(49,59] 13.35802 703.02068 0.019 0.985

Age(59,69] 12.81833 703.02054 0.018 0.985

Age(69,79] 13.34867 703.02059 0.019 0.985

Age(79,89] 13.59651 703.02058 0.019 0.985

Age(89,104] 13.84135 703.02059 0.020 0.984

CACI1 -26.45192 832.07094 -0.032 0.975

CACI2 -12.50378 703.02110 -0.018 0.986

CACI3 -12.38379 703.02137 -0.018 0.986

CACI4 -12.20188 703.02130 -0.017 0.986

CACI5 -12.08901 703.02131 -0.017 0.986

CACI6 -12.03757 703.02131 -0.017 0.986

CACI7 -11.61291 703.02132 -0.017 0.987

CACI8 -11.94812 703.02135 -0.017 0.986

CACI9 -12.00075 703.02138 -0.017 0.986

CACI10 -11.92137 703.02144 -0.017 0.986

CACI11 -12.34551 703.02159 -0.018 0.986

CACI12 -10.89865 703.02150 -0.016 0.988

CACI13 -11.48901 703.02206 -0.016 0.987

CACI14 -27.72859 3223.55443 -0.009 0.993

CACI15 -28.31136 2817.31415 -0.010 0.992

CACI16 -26.73763 6560.41564 -0.004 0.997

CACI17 -10.68905 703.02209 -0.015 0.988

CACI18 -26.09065 6560.41564 -0.004 0.997

CACI20 -27.28373 6560.41563 -0.004 0.997

CACI21 5.17659 6560.41563 0.001 0.999

CACI28 7.21739 6560.41563 0.001 0.999

---

Signif. codes: 0 ‘***’ 0.001 ‘**’ 0.01 ‘*’ 0.05 ‘.’ 0.1 ‘ ’ 1

**Multivariate analysis for NEWS2, age, sex and Charlson Age-Comorbidity Index at 2-days mortality**

Coefficients:

Estimate Std. Error z value Pr(>|z|)

(Intercept) -5.75052 0.72696 -7.910 2.57e-15 ***

NEWS2 0.30855 0.02105 14.659 < 2e-16 ***

Sex1 0.02481 0.15390 0.161 0.872

Age(49,59] 13.35594 692.96050 0.019 0.985

Age(59,69] 13.25043 692.96031 0.019 0.985

Age(69,79] 13.71478 692.96036 0.020 0.984

Age(79,89] 14.21449 692.96035 0.021 0.984

Age(89,104] 14.38077 692.96036 0.021 0.983

CACI1 -27.14902 821.86354 -0.033 0.974

CACI2 -13.22677 692.96057 -0.019 0.985

CACI3 -13.48919 692.96081 -0.019 0.984

CACI4 -12.70278 692.96072 -0.018 0.985

CACI5 -12.96890 692.96073 -0.019 0.985

CACI6 -12.72250 692.96073 -0.018 0.985

CACI7 -12.61339 692.96074 -0.018 0.985

CACI8 -12.73831 692.96075 -0.018 0.985

CACI9 -12.85109 692.96078 -0.019 0.985

CACI10 -12.85806 692.96083 -0.019 0.985

CACI11 -13.66526 692.96102 -0.020 0.984

CACI12 -11.60087 692.96089 -0.017 0.987

CACI13 -11.77982 692.96139 -0.017 0.986

CACI14 -28.98317 3183.86575 -0.009 0.993

CACI15 -12.32916 692.96184 -0.018 0.986

CACI16 -27.78934 6559.34516 -0.004 0.997

CACI17 -11.97564 692.96153 -0.017 0.986

CACI18 -27.36341 6559.34516 -0.004 0.997

CACI20 -28.59760 6559.34516 -0.004 0.997

CACI21 3.76487 6559.34516 0.001 1.000

CACI28 5.89989 6559.34515 0.001 0.999

---

Signif. codes: 0 ‘***’ 0.001 ‘**’ 0.01 ‘*’ 0.05 ‘.’ 0.1 ‘ ’ 1

**Multivariate analysis for NEWS2, age, sex and Charlson Age-Comorbidity Index at 7-days mortality**

Coefficients:

Estimate Std. Error z value Pr(>|z|)

(Intercept) -5.78282 0.72187 -8.011 1.14e-15 ***

NEWS2 0.28173 0.01609 17.505 < 2e-16 ***

Sex1 0.30564 0.11435 2.673 0.00752 **

Age(49,59] 0.33468 1.13126 0.296 0.76735

Age(59,69] 0.48198 1.13848 0.423 0.67204

Age(69,79] 1.42857 1.14444 1.248 0.21193

Age(79,89] 2.04606 1.14335 1.790 0.07353 .

Age(89,104] 2.24910 1.14560 1.963 0.04962 *

CACI1 -0.46938 1.34319 -0.349 0.72675

CACI2 0.35131 1.33128 0.264 0.79187

CACI3 0.22792 1.36464 0.167 0.86735

CACI4 0.80183 1.34814 0.595 0.55200

CACI5 0.63050 1.35127 0.467 0.64079

CACI6 0.78881 1.35137 0.584 0.55941

CACI7 0.63605 1.35615 0.469 0.63906

CACI8 0.79824 1.35843 0.588 0.55679

CACI9 0.90238 1.36464 0.661 0.50845

CACI10 0.57831 1.37966 0.419 0.67509

CACI11 -0.42875 1.43585 -0.299 0.76524

CACI12 1.19504 1.42406 0.839 0.40137

CACI13 0.51984 1.63847 0.317 0.75104

CACI14 -12.63502 412.20616 -0.031 0.97555

CACI15 1.98856 1.65984 1.198 0.23090

CACI16 -11.64437 882.74441 -0.013 0.98948

CACI17 0.40193 1.70558 0.236 0.81370

CACI18 -11.41667 882.74441 -0.013 0.98968

CACI20 -12.54359 882.74441 -0.014 0.98866

CACI21 12.46519 882.74445 0.014 0.98873

CACI28 14.13166 882.74443 0.016 0.98723

---

Signif. codes: 0 ‘***’ 0.001 ‘**’ 0.01 ‘*’ 0.05 ‘.’ 0.1 ‘ ’ 1

**Multivariate analysis for NEWS2, age, sex and Charlson Age-Comorbidity Index at 14-days mortality**

Coefficients:

Estimate Std. Error z value Pr(>|z|)

(Intercept) -5.7495 0.7204 -7.981 1.45e-15 ***

NEWS2 0.2713 0.0150 18.088 < 2e-16 ***

Sex1 0.3305 0.1052 3.141 0.00168 **

Age(49,59] -0.5457 0.7381 -0.739 0.45970

Age(59,69] -0.2505 0.7458 -0.336 0.73697

Age(69,79] 0.6012 0.7503 0.801 0.42300

Age(79,89] 1.3713 0.7494 1.830 0.06728 .

Age(89,104] 1.6402 0.7525 2.180 0.02928 *

CACI1 0.9032 1.0045 0.899 0.36860

CACI2 1.4438 1.0236 1.410 0.15841

CACI3 1.5932 1.0484 1.520 0.12861

CACI4 1.9249 1.0347 1.860 0.06283 .

CACI5 1.8957 1.0375 1.827 0.06766 .

CACI6 1.8737 1.0386 1.804 0.07120 .

CACI7 2.0600 1.0426 1.976 0.04816 *

CACI8 1.8695 1.0466 1.786 0.07406 .

CACI9 2.0711 1.0540 1.965 0.04940 *

CACI10 1.8288 1.0681 1.712 0.08685 .

CACI11 1.7110 1.1029 1.551 0.12081

CACI12 2.3467 1.1237 2.088 0.03677 *

CACI13 1.2556 1.3918 0.902 0.36699

CACI14 -11.8481 404.7027 -0.029 0.97664

CACI15 3.7365 1.5455 2.418 0.01562 *

CACI16 -10.8336 882.7440 -0.012 0.99021

CACI17 2.3411 1.5794 1.482 0.13828

CACI18 -10.7898 882.7440 -0.012 0.99025

CACI20 -11.8751 882.7440 -0.013 0.98927

CACI21 13.2488 882.7440 0.015 0.98803

CACI28 14.8175 882.7440 0.017 0.98661

---

Signif. codes: 0 ‘***’ 0.001 ‘**’ 0.01 ‘*’ 0.05 ‘.’ 0.1 ‘ ’ 1

**Multivariate analysis for NEWS2, age, sex and Charlson Age-Comorbidity Index at 30-days mortality**

Coefficients:

Estimate Std. Error z value Pr(>|z|)

(Intercept) -5.39883 0.59386 -9.091 < 2e-16 ***

NEWS2 0.27812 0.01484 18.745 < 2e-16 ***

Sex1 0.38623 0.10221 3.779 0.000158 ***

Age(49,59] -0.62966 0.65862 -0.956 0.339060

Age(59,69] -0.21154 0.66095 -0.320 0.748926

Age(69,79] 0.53508 0.66750 0.802 0.422774

Age(79,89] 1.31392 0.66710 1.970 0.048884 *

Age(89,104] 1.64277 0.67062 2.450 0.014300 *

CACI1 0.77189 0.86298 0.894 0.371080

CACI2 1.43704 0.87452 1.643 0.100337

CACI3 1.50037 0.90027 1.667 0.095600 .

CACI4 1.80053 0.88735 2.029 0.042446 *

CACI5 1.75227 0.89039 1.968 0.049069 *

CACI6 1.75373 0.89158 1.967 0.049183 *

CACI7 1.93650 0.89620 2.161 0.030711 *

CACI8 1.80003 0.90028 1.999 0.045565 *

CACI9 1.99865 0.90925 2.198 0.027939 *

CACI10 1.72159 0.92400 1.863 0.062434 .

CACI11 1.45544 0.96498 1.508 0.131490

CACI12 1.96017 0.99064 1.979 0.047851 *

CACI13 2.31036 1.16831 1.978 0.047982 *

CACI14 -12.19986 400.65246 -0.030 0.975708

CACI15 3.36143 1.45210 2.315 0.020619 *

CACI16 -11.20100 882.74382 -0.013 0.989876

CACI17 1.91871 1.48830 1.289 0.197331

CACI18 -11.14550 882.74382 -0.013 0.989926

CACI20 -12.25796 882.74382 -0.014 0.988921

CACI21 12.75983 882.74386 0.014 0.988467

CACI28 14.32041 882.74384 0.016 0.987057

---

Signif. codes: 0 ‘***’ 0.001 ‘**’ 0.01 ‘*’ 0.05 ‘.’ 0.1 ‘ ’ 1

**Multivariate analysis for NEWS2, age, sex and Charlson Age-Comorbidity Index at 90-days mortality**

Coefficients:

Estimate Std. Error z value Pr(>|z|)

(Intercept) -5.43808 0.59412 -9.153 < 2e-16 ***

NEWS2 0.28283 0.01475 19.178 < 2e-16 ***

Sex1 0.41439 0.09954 4.163 3.14e-05 ***

Age(49,59] -1.15243 0.54865 -2.100 0.035686 *

Age(59,69] -0.61406 0.55388 -1.109 0.267579

Age(69,79] 0.10314 0.55619 0.185 0.852886

Age(79,89] 0.74481 0.55614 1.339 0.180492

Age(89,104] 1.10532 0.56043 1.972 0.048578 *

CACI1 1.62629 0.77412 2.101 0.035656 *

CACI2 1.88811 0.80348 2.350 0.018778 *

CACI3 2.30269 0.81858 2.813 0.004907 **

CACI4 2.48157 0.80736 3.074 0.002114 **

CACI5 2.53879 0.81007 3.134 0.001724 **

CACI6 2.62555 0.81111 3.237 0.001208 **

CACI7 2.72758 0.81653 3.340 0.000836 ***

CACI8 2.63244 0.82071 3.208 0.001339 **

CACI9 2.71422 0.83125 3.265 0.001094 **

CACI10 2.59258 0.84558 3.066 0.002169 **

CACI11 2.40055 0.88760 2.705 0.006840 **

CACI12 3.23226 0.91281 3.541 0.000399 ***

CACI13 3.33467 1.06827 3.122 0.001799 **

CACI14 1.69367 1.45208 1.166 0.243463

CACI15 3.92089 1.40516 2.790 0.005265 **

CACI16 -9.77683 535.41178 -0.018 0.985431

CACI17 2.40066 1.43832 1.669 0.095104 .

CACI18 -9.57002 535.41178 -0.018 0.985739

CACI20 -10.70133 535.41178 -0.020 0.984054

CACI21 12.24225 535.41183 0.023 0.981758

CACI28 13.80766 535.41180 0.026 0.979426

---

Signif. codes: 0 ‘***’ 0.001 ‘**’ 0.01 ‘*’ 0.05 ‘.’ 0.1 ‘ ’ 1

**Multivariate analysis for qCSI, age, sex and Charlson Age-Comorbidity Index at 1-days mortality**

Coefficients:

Estimate Std. Error z value Pr(>|z|)

(Intercept) -5.54194 1.00933 -5.491 4e-08 ***

qCSI 0.25901 0.02470 10.485 <2e-16 ***

Sex1 -0.05233 0.17355 -0.302 0.763

Age(49,59] 13.80057 733.03126 0.019 0.985

Age(59,69] 13.06059 733.03113 0.018 0.986

Age(69,79] 13.62636 733.03113 0.019 0.985

Age(79,89] 13.91032 733.03113 0.019 0.985

Age(89,104] 14.20065 733.03113 0.019 0.985

CACI1 -26.87768 865.04674 -0.031 0.975

CACI2 -12.63427 733.03177 -0.017 0.986

CACI3 -12.42703 733.03189 -0.017 0.986

CACI4 -12.43222 733.03181 -0.017 0.986

CACI5 -12.02395 733.03183 -0.016 0.987

CACI6 -11.97082 733.03183 -0.016 0.987

CACI7 -11.58115 733.03184 -0.016 0.987

CACI8 -11.81738 733.03186 -0.016 0.987

CACI9 -12.01979 733.03190 -0.016 0.987

CACI10 -11.78754 733.03194 -0.016 0.987

CACI11 -12.05477 733.03208 -0.016 0.987

CACI12 -10.93069 733.03198 -0.015 0.988

CACI13 -11.02605 733.03231 -0.015 0.988

CACI14 -27.47445 3230.31385 -0.009 0.993

CACI15 -28.12406 2711.11639 -0.010 0.992

CACI16 -27.11618 6563.69941 -0.004 0.997

CACI17 -10.49241 733.03257 -0.014 0.989

CACI18 -26.88212 6563.69941 -0.004 0.997

CACI20 -26.88212 6563.69941 -0.004 0.997

CACI21 7.05826 6563.69941 0.001 0.999

CACI28 7.36960 6563.69942 0.001 0.999

---

Signif. codes: 0 ‘***’ 0.001 ‘**’ 0.01 ‘*’ 0.05 ‘.’ 0.1 ‘ ’ 1

**Multivariate analysis for qCSI, age, sex and Charlson Age-Comorbidity Index at 2-days mortality**

Coefficients:

Estimate Std. Error z value Pr(>|z|)

(Intercept) -4.87359 0.72009 -6.768 1.31e-11 ***

qCSI 0.28635 0.02193 13.060 < 2e-16 ***

Sex1 -0.07975 0.15051 -0.530 0.596

Age(49,59] 13.87688 719.30848 0.019 0.985

Age(59,69] 13.49385 719.30832 0.019 0.985

Age(69,79] 14.03801 719.30832 0.020 0.984

Age(79,89] 14.53697 719.30831 0.020 0.984

Age(89,104] 14.75314 719.30832 0.021 0.984

CACI1 -27.65216 851.24744 -0.032 0.974

CACI2 -13.41635 719.30866 -0.019 0.985

CACI3 -13.61234 719.30876 -0.019 0.985

CACI4 -13.05698 719.30866 -0.018 0.986

CACI5 -12.99641 719.30868 -0.018 0.986

CACI6 -12.74274 719.30868 -0.018 0.986

CACI7 -12.65921 719.30869 -0.018 0.986

CACI8 -12.70868 719.30870 -0.018 0.986

CACI9 -12.95565 719.30873 -0.018 0.986

CACI10 -12.79580 719.30877 -0.018 0.986

CACI11 -13.43177 719.30895 -0.019 0.985

CACI12 -11.78534 719.30881 -0.016 0.987

CACI13 -11.67223 719.30909 -0.016 0.987

CACI14 -28.71204 3214.48538 -0.009 0.993

CACI15 -12.06267 719.30998 -0.017 0.987

CACI16 -28.22344 6562.18099 -0.004 0.997

CACI17 -11.89907 719.30944 -0.017 0.987

CACI18 -28.14970 6562.18099 -0.004 0.997

CACI20 -28.14970 6562.18099 -0.004 0.997

CACI21 5.53668 6562.18098 0.001 0.999

CACI28 5.90278 6562.18098 0.001 0.999

---

Signif. codes: 0 ‘***’ 0.001 ‘**’ 0.01 ‘*’ 0.05 ‘.’ 0.1 ‘ ’ 1

**Multivariate analysis for qCSI, age, sex and Charlson Age-Comorbidity Index at 7-days mortality**

Coefficients:

Estimate Std. Error z value Pr(>|z|)

(Intercept) -5.017606 0.719293 -6.976 3.04e-12 ***

qCSI 0.279479 0.017358 16.101 < 2e-16 ***

Sex1 0.209038 0.112082 1.865 0.0622 .

Age(49,59] 0.479422 1.116882 0.429 0.6677

Age(59,69] 0.419404 1.106724 0.379 0.7047

Age(69,79] 1.418070 1.107757 1.280 0.2005

Age(79,89] 2.002597 1.106422 1.810 0.0703 .

Age(89,104] 2.247327 1.108473 2.027 0.0426 *

CACI1 -0.504955 1.369216 -0.369 0.7123

CACI2 0.424618 1.313099 0.323 0.7464

CACI3 0.318426 1.334791 0.239 0.8114

CACI4 0.714571 1.316856 0.543 0.5874

CACI5 0.806032 1.320508 0.610 0.5416

CACI6 1.010034 1.321132 0.765 0.4446

CACI7 0.833391 1.325162 0.629 0.5294

CACI8 1.040390 1.327644 0.784 0.4333

CACI9 1.072719 1.334899 0.804 0.4216

CACI10 0.851606 1.347585 0.632 0.5274

CACI11 -0.003756 1.401976 -0.003 0.9979

CACI12 1.231375 1.385729 0.889 0.3742

CACI13 0.920899 1.524004 0.604 0.5457

CACI14 -12.121840 415.921678 -0.029 0.9767

CACI15 2.831920 1.644660 1.722 0.0851 .

CACI16 -11.734528 882.744362 -0.013 0.9894

CACI17 0.698704 1.682670 0.415 0.6780

CACI18 -11.760097 882.744361 -0.013 0.9894

CACI20 -11.760097 882.744361 -0.013 0.9894

CACI21 14.262074 882.744379 0.016 0.9871

CACI28 14.332515 882.744379 0.016 0.9870

---

Signif. codes: 0 ‘***’ 0.001 ‘**’ 0.01 ‘*’ 0.05 ‘.’ 0.1 ‘ ’ 1

**Multivariate analysis for qCSI, age, sex and Charlson Age-Comorbidity Index at 14-days mortality**

Coefficients:

Estimate Std. Error z value Pr(>|z|)

(Intercept) -5.00800 0.71776 -6.977 3.01e-12 ***

qCSI 0.26421 0.01625 16.260 < 2e-16 ***

Sex1 0.24181 0.10275 2.353 0.01861 *

Age(49,59] -0.44107 0.72326 -0.610 0.54197

Age(59,69] -0.28894 0.71711 -0.403 0.68701

Age(69,79] 0.59883 0.71821 0.834 0.40441

Age(79,89] 1.32656 0.71707 1.850 0.06432 .

Age(89,104] 1.62574 0.71998 2.258 0.02394 *

CACI1 0.93146 1.01480 0.918 0.35868

CACI2 1.50624 1.00865 1.493 0.13535

CACI3 1.66699 1.02595 1.625 0.10420

CACI4 1.84227 1.01162 1.821 0.06859 .

CACI5 2.04508 1.01460 2.016 0.04384 *

CACI6 2.08587 1.01609 2.053 0.04009 *

CACI7 2.23477 1.01952 2.192 0.02838 *

CACI8 2.10068 1.02358 2.052 0.04014 *

CACI9 2.23628 1.03151 2.168 0.03016 *

CACI10 2.06900 1.04369 1.982 0.04744 *

CACI11 2.05232 1.07756 1.905 0.05683 .

CACI12 2.34640 1.09404 2.145 0.03198 *

CACI13 1.67867 1.26628 1.326 0.18495

CACI14 -11.31818 415.33359 -0.027 0.97826

CACI15 4.56419 1.53363 2.976 0.00292 **

CACI16 -10.92712 882.74396 -0.012 0.99012

CACI17 2.64660 1.55836 1.698 0.08945 .

CACI18 -11.12643 882.74396 -0.013 0.98994

CACI20 -11.12643 882.74396 -0.013 0.98994

CACI21 15.04201 882.74397 0.017 0.98640

CACI28 15.06442 882.74397 0.017 0.98638

---

Signif. codes: 0 ‘***’ 0.001 ‘**’ 0.01 ‘*’ 0.05 ‘.’ 0.1 ‘ ’ 1

**Multivariate analysis for qCSI, age, sex and Charlson Age-Comorbidity Index at 30-days mortality**

Coefficients:

Estimate Std. Error z value Pr(>|z|)

(Intercept) -4.64224 0.59098 -7.855 3.99e-15 ***

qCSI 0.27527 0.01611 17.088 < 2e-16 ***

Sex1 0.29472 0.09981 2.953 0.00315 **

Age(49,59] -0.52351 0.64506 -0.812 0.41704

Age(59,69] -0.25275 0.63635 -0.397 0.69123

Age(69,79] 0.52906 0.64035 0.826 0.40869

Age(79,89] 1.26199 0.63968 1.973 0.04851 *

Age(89,104] 1.61606 0.64302 2.513 0.01196 *

CACI1 0.79583 0.87080 0.914 0.36077

CACI2 1.47909 0.86070 1.718 0.08571 .

CACI3 1.55782 0.88090 1.768 0.07699 .

CACI4 1.70122 0.86748 1.961 0.04987 *

CACI5 1.88678 0.87061 2.167 0.03022 *

CACI6 1.95571 0.87223 2.242 0.02495 *

CACI7 2.09949 0.87630 2.396 0.01658 *

CACI8 2.02191 0.88038 2.297 0.02164 *

CACI9 2.15611 0.88953 2.424 0.01536 *

CACI10 1.94765 0.90243 2.158 0.03091 *

CACI11 1.79402 0.94293 1.903 0.05709 .

CACI12 1.94215 0.96352 2.016 0.04383 *

CACI13 2.30971 1.09589 2.108 0.03506 *

CACI14 -11.67973 410.67986 -0.028 0.97731

CACI15 4.21981 1.44394 2.922 0.00347 **

CACI16 -11.29814 882.74380 -0.013 0.98979

CACI17 2.21089 1.47101 1.503 0.13284

CACI18 -11.48055 882.74380 -0.013 0.98962

CACI20 -11.48055 882.74380 -0.013 0.98962

CACI21 14.56432 882.74382 0.016 0.98684

CACI28 14.54487 882.74382 0.016 0.98685

---

Signif. codes: 0 ‘***’ 0.001 ‘**’ 0.01 ‘*’ 0.05 ‘.’ 0.1 ‘ ’ 1

**Multivariate analysis for qCSI, age, sex and Charlson Age-Comorbidity Index at 90-days mortality**

Coefficients:

Estimate Std. Error z value Pr(>|z|)

(Intercept) -4.66238 0.59125 -7.886 3.13e-15 ***

qCSI 0.27830 0.01599 17.406 < 2e-16 ***

Sex1 0.31930 0.09704 3.290 0.001000 **

Age(49,59] -1.04399 0.53559 -1.949 0.051267 .

Age(59,69] -0.63785 0.53396 -1.195 0.232264

Age(69,79] 0.10382 0.53496 0.194 0.846116

Age(79,89] 0.70269 0.53481 1.314 0.188875

Age(89,104] 1.08464 0.53896 2.012 0.044173 *

CACI1 1.65619 0.77568 2.135 0.032748 *

CACI2 1.91614 0.79276 2.417 0.015647 *

CACI3 2.33914 0.80465 2.907 0.003649 **

CACI4 2.37096 0.79356 2.988 0.002810 **

CACI5 2.64876 0.79622 3.327 0.000879 ***

CACI6 2.80628 0.79779 3.518 0.000436 ***

CACI7 2.87341 0.80258 3.580 0.000343 ***

CACI8 2.83728 0.80671 3.517 0.000436 ***

CACI9 2.85885 0.81700 3.499 0.000467 ***

CACI10 2.78863 0.82955 3.362 0.000775 ***

CACI11 2.71886 0.87162 3.119 0.001813 **

CACI12 3.14748 0.89624 3.512 0.000445 ***

CACI13 3.22375 1.01991 3.161 0.001573 **

CACI14 2.29233 1.46490 1.565 0.117622

CACI15 4.78031 1.40150 3.411 0.000648 ***

CACI16 -9.88341 535.41176 -0.018 0.985272

CACI17 2.71337 1.43129 1.896 0.057992 .

CACI18 -9.92568 535.41176 -0.019 0.985209

CACI20 -9.92568 535.41176 -0.019 0.985209

CACI21 14.08250 535.41178 0.026 0.979016

CACI28 14.04150 535.41178 0.026 0.979077

---

Signif. codes: 0 ‘***’ 0.001 ‘**’ 0.01 ‘*’ 0.05 ‘.’ 0.1 ‘ ’ 1
